# Supplementary figures and images for: Inflammatory Microenvironment in Early Non-Small Cell Lung Cancer: Exploring the Predictive Value of Radiomics
Source: Cancers (Basel). 2022 Jul 8;14(14):3335. doi: 10.3390/cancers14143335 (PMC9323656; doi:10.3390/cancers14143335)

Supplementary

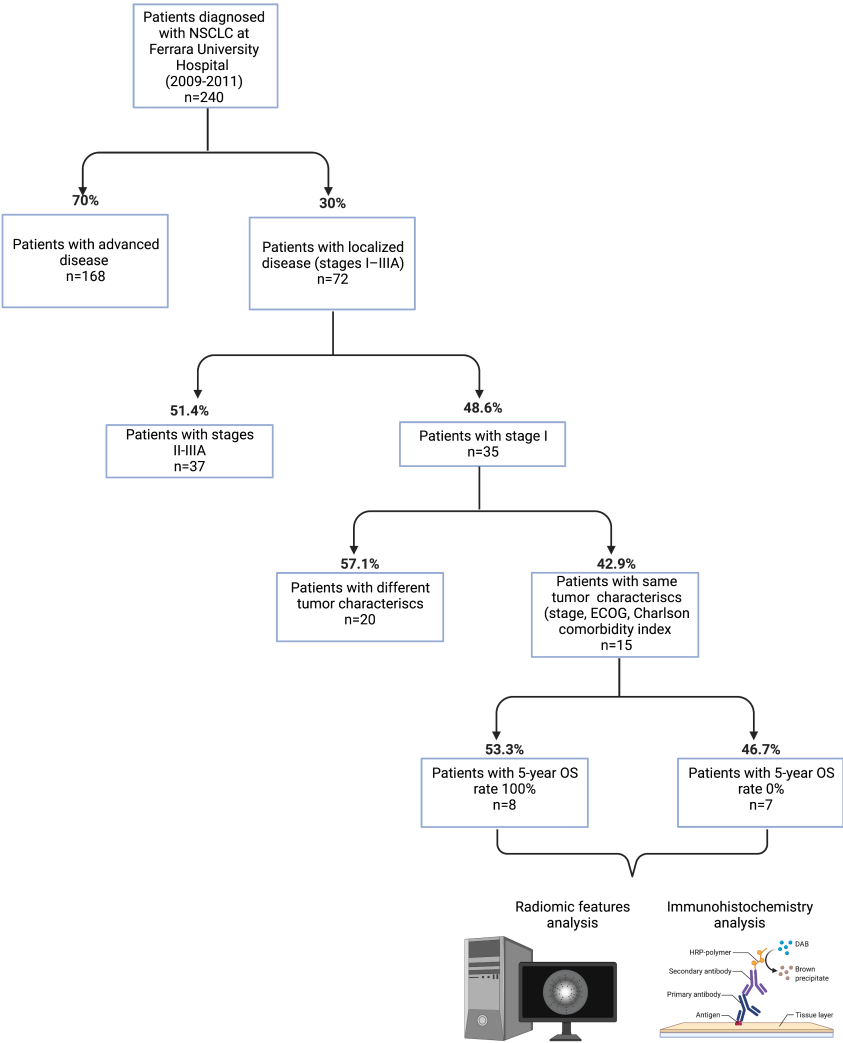

Supplement: Supplementary file 1 [file cancers-14-03335-s001.zip › cancers-1789451-supplementary.pdf]
